# Periodontitis as a Risk Factor in Non-Diabetic Patients with Coronary Artery Disease

**Published:** 2010

**Authors:** Vida Nesarhoseini, Mahmoud khosravi

**Affiliations:** 1 MD, Department of Cardiology, Mazandaran University of Medical Science, Sari, Iran; 2 Department of Dental Periodontology, Babol University of Dentis, Sari, Iran

**Keywords:** Coronary artery disease (CAD), Gingival. inflammation (periodontitis), Coronary angiography

## Abstract

**BACKGROUND:**

Coronary artery disease (CAD) is responsible for many mortality across the world, especially in our country.The conventional risk factors for atherosclerosis are well understood, but they can account for only about50% to 70% of atherosclerotic events in the general population. The aim of this study was to investigate relationships between prevalent coronary artery disease(CAD) and clinical periodontal disease in patients with angiographic ally proven coronary artery disease.

**METHODS:**

152 consecutive patients with angiographically proven coronary artery disease will be included in this study, who received a complete periodontal examination during visit.

**RESULTS:**

Patients with normal coronary, average plaque index (1.6±1.02) Index of bleeding (1.51±0.92),mean adhesion level (3.57±1.18). But patients with coronary artery disease, the mean plaque index (2.46±0.62) Index of bleeding (1.86±0.92), mean adhesion level (4.13±1.45). This differences are statistically significant. (P <0.05) In this study, average depth of probe entrance on the surface of teeth has had little relation with cardiovascular disease (p=0.051).

**CONCLUSION:**

According to the results of this study, in peoples over 40 years, who had coronary artery disease proved by coronary angiography, gingival inflammation (periodentitis) has a significant relation as a risk factor.

## Introduction

Coronary artery disease (CAD) remains the principal cause of death in most countries, despite significant preventive and therapeutic advances. It has many known risk factors like, Hypertension, Hyperlipidemia, Diabetes mellitus, Positive Family history, Smoking and so on. But many conditions increase risk of CAD yet, through atherosclerosis.^1^, ^3^

Recent studies illustrate the existence of a relation between periodontal disorders and coronary artery disease, which power the probable effect of periodontal disease as a risk factor for(CAD.^4^, ^5^ Otherwise another were experienced insignificant relation between (CAD) and periodentitis(8-10). Periodontitis is associated with endotoxemia, leakage of lipopolysaccharides ( LPS)deriving from periodontal pathogens into circulation(4,20). LPS is one of the potent stimulators of systemic inflammation and intima wall macrophage-derived foam cell formation, and therefore it is considered a proatherogenic compound, through the response to increasing levels of acute phase proteins (CRP).^7^, ^8^, ^9^

Also recent epidemiologic studies show that high CRP as a risk factor is considered for cardiovascular events.^10^ Also, an intervention study statement on whether the treatment of gingival inflammation (periodentitis) leads to reduced CHD mortality is not done.^6^

## Materials and Method

A cohort study was done on 152 patients referring to Mazandaran Heart Center in North of Iran. Inclusion criteria: Age over the 40 years who's Coronary artery disease as defined by previous or current detection of 50% stenosis of a main coronary artery by coronary angiography.Or no significant stenosis of coronary artery.

Exclusion criteria: Diabetic, Periodontal treatment and/or antibiotic therapy during the last 6 months, Pregnancy, Current alcohol or drug abuse, or psychological reasons that make study participation impractical

Drugs which are potential causal for gingival hyperplasia such as (Hydantoin, Nifedipine, Cyclosporin A, and other)

The people studied divided in two groups by coronary angiography results.. Demographic information were derived from questions asked during the interviewed to age, sex, literacy level, weight, LDL and HDL, exercise,, smoking, blood pressure for all the two groups. Then a periodontal examination was done (by general dentist and periodontitis) for all participants of the study, who was unaware from the result of patient's angiography.

Coronary artery disease defined by stenosis more than 50% lumen in at least one coronary artery in angiography.Periodontal disease is an inflammatory disease of tissues or teeth holder tissue that gradually causes the destruction of tissues and loss of teeth. Clinical periodontal examination included measuring plaque (plaque terms), bleeding on examination with the probe (Barnett bleeding indexes), Probing packet depth at the mesial, distal, Bucal, Palatal or Lingual surface of all teeth except the third molar has been done and CAL (Clinical Attachment Level) was calculated.

Plaques were recorded according to Silness & loe index. Plaque depth measuring, the entrance depth of probe in longitudinal axis of tooth and also CAL as mm is registered and the number of teeth remaining were recorded.

Plaque index (Silness & loe): accumulation of debries in gingival margins of tooth that is determined with the scale of 0 to 3.

0 = No plaque

1 =A film of plaque adhering to the free gingival margin and adjacent area of the tooth. The plaque may be observed in situ only after application of a disclosing solution or by using a probe on the tooth surface.

2 = moderate accumulation of soft deposits within gingival pocket, or on the tooth and gingival margin, that can be seen with the naked eye.

3 = an abundance of soft matter within the gingival pocket, on the tooth and gingival margin, in all these areas.

Modified papillary Bleeding Index (Barnett) bleeding after the probing of gums sulcus bleeding gums, diffuse marginal inflammation, and swollen red papillae is determined with the Scale of zero to 3:

Zero: the lack of bleeding after 30 seconds

One: bleeding after 30 seconds

Two: bleeding 2 to 30 seconds

Three: bleeding less than 2 seconds

Gingival groove depth: Shallow crevice or space around the tooth bounded by the surface of the tooth on one side and the epithelium lining the free margin of the gingiva on the other, V shaped. Sulcus depth can be measured by a periodontal probe.Histologic depth is about 1.8mm, probing depth is 2–3 mm.

Clinical Attachment Level: The amount of space between attached periodontal tissues and a fixed point, usually the cement enamel junction.

A measurement used to assess the stability of attachment as part of a periodontal maintenance program.

Statistical significance was set at 0.05, and the unit of analysis was the person.. Bivariate relationships were assessed by *t* tests or Kolmogorov-Smirnov tests for continuous variables and Cochran Mantel-Haenszel χ^2^ statistics and odds ratios and 95% CIs for categorical variables.. Potential confounders were based on the literature and our previous findings on the relationship between clinical periodontal disease and CAD.^13^–^20^

## Results

152 patients were included in this study.

There were 54.6% (83)men and the 45.4% (69) were female. The mean age for case group was 51.1+/-7.3(mean+/-SD) and 51.3+/-10.3 years for control group. In male participants, 37 patients (44.6%) had coronary artery disease and among women 39 cases (56.5%) had CAD, which sex difference was not significant (p= 0.96) (Table 1).

**Table 1:** distribution of people with coronary heart disease and without coronary heart disease according to gender

CHD Gender	Patients With CHD (percent)	Patients Without CHD(percent)	Total
Male	37	46	83
	(44.6)	(55.4)	
Women	39	30	69
	(56.5)	(43.5)	
Illiterate or elementary	51	25	76
	(67.1)	(32.9)	
Guidance school	10	13	23
	(43.5)	(56.5)	
High School	11	14	25
	(44)	(56)	
Higher diploma	4	24	28
	(14.3)	(85.7)	

The level of education and physical activity, has contrary effect on CAD and this difference was statistically significant (p <0.05). (Table 2,)

**Table 2:** Distribution of people with coronary heart disease and without coronary heart disease according to sport

CHD			
Exercise	Number of people With CHD (percent)	Number of people Without CHD (percent)	Total
Loss of Physical activity	66	24	90
	(73.3)	(26.7)	
Regular exercise	1	32	33
	(3)	(97)	
Irregular exercise	9	20	29
	(31)	(69)	

**Table 3:** distribution of people with coronary heart disease without coronary heart disease by smoking

CHD			
Cigarettes	Patients With CHD (percent)	Patients Without CHD (percent)	Total
Smoking	32	8	40
	(80)	(20)	
Non-smoking	44	68	112
	(39.3)	(60.7)	

**Table 4:** -distribution of people with coronary heart disease without coronary heart disease based on HPL

CHD			
Hyperlipidemi(TC,LDL)	Numbe r of people With CHD (percent)	Number of people Without CHD (percent)	Total
TC>250	34	10	
LDL>180	(77.3)	(22.7)	44
TC<250	42	66	108
LDL<180	(38.9)	(61.1)	

**Table 5:** distribution of people with coronary heart disease without coronary heart disease based on history of hypertension

Heart disease			
Hypertension	Number of people With heart disease (percent)	Number of people Without heart disease (percent)	Total
History of hypertension	51	9	60
	(85)	(15)	
Without History of hypertension	25	67	92
	(27.2)	(72.8)	

The gingival index( GI) average was higher in patients with CAD (70.2%) than control group(29.8%),as like Bleeding index(BI) and this difference is statistically significant. (P <0.05) (Table 6, 7)

**Table 6:** distribution of people with coronary heart disease and coronary heart disease based on GI.

HD			
GI	Number of people With CHD (percent)	Number of people Without CHD (percent)	Total
Score 0	0	13	13
	(0)	(100)	
Score 1	5	21	26
	(19.2)	(80.8)	
Score 2	31	25	56
	(55.4)	(44.6)	
Score 3	40	17	57
	(70.2)	(29.8)	

**Table 7:** Distribution individuals with coronary heart disease and coronary heart disease according to Index of bleeding

Heart disease			
Bleeding index	Number of people With CHD (percent)	Number of people Without CHD (percent)	Total
Score 0	7	18	25
	(28)	(72)	
Score 1	17	19	36
	(47.2)	(52.8)	
Score 2	31	21	52
	(59.6)	(40.46)	
Score 3	21	18	39
	(53.8)	(46.2)	

**Table 8:** Distribution of individuals with coronary heart disease without coronary heart disease based on depth of Probe entrance

Heart disease			
Entrance depth of probe	Number of people With CHD (percent)	Number of people Without CHD (percent)	Total
2 mm	8	12	20
	(40)	(60)	
3 mm	20	24	44
	(45.5)	(54.5)	
4 mm	14	20	34
	(41.2)	(58.8)	
5 mm	6	5	11
	(54.5)	(45.5)	
6 mm	9	2	11
	(81.8)	(18.2)	
7 mm	13	7	20
	(65)	(35)	
8 mm	6	6	12
	(50)	(50)	

The relationship between Entrance depth of probe and CAD was not statistically significant. P = 0.5 (Table 9)

**Table 9:** Distribution of individuals with coronary heart disease and without coronary heart disease based on the amount of clinical adhesion.

CAL			
clinical attachment	Number of people With CHD (percent)	Number of people Without CHD (percent)	Total
1 mm	0	1	1
	(0)	(100)	
2 mm	10	13	23
	(43.5)	(56.5)	
3 mm	20	34	54
	(37)	(63)	
4 mm	16	10	26
	(61.5)	(38.5)	
5 mm	15	8	23
	(65.2)	(34.8)	
6 mm	10	7	17
	(58.8)	(41.2)	

Coefficient of plaque index with entrance depth of Probe is 0.659 that is statistically significant (P <0.05)

Coefficient of plaque index with clinical adhesion rate is 0.664 that is statistically significant (p<0.05)

Coefficient of bleeding index with entrance depth of probe is 0.685 that is statistically significant (p<0.05)

Coefficient of bleeding index with clinical adhesion rate is 0.686 that is statistically significant (p<0.05)

Coefficient of entrance depth of probe with clinical adhesion rate is 0.894 that is statistically significant (p<0.05).

Measurement of clinical attachment level more likely reflects periodontal disease. The statistically significant difference was found in the clinical attachment level (p<0.005), where a higher mean value was in patients with coronary artery disease (53.8%) compared with patients without CAD (46.2%).(Table 10)

## Conclusion

This study suggests a possible association between Periodontitis and CAD.

Since 3 main indices out of 4 indices for periodontal diseases such as swollen red papillae, bleeding gums, or diffuse marginal inflammation, correlated with increased risk of coronary artery disease in our research and most other studies, periodontal disease may be regarded as an independent risk factor for coronary artery disease.

## Discussion

The present study demonstrated higher abnormal Periodontal Indices in patients with coronary artery disease than normal groups as independent risk factor.

Several theories exist to explain the link between periodontal disease and heart disease. One theory is that oral bacteria can affect the heart when they enter the blood stream, attaching to fatty plaques in the coronary arteries (heart blood vessels) and contributing to clot formation. Coronary artery disease is characterized by a thickening of the walls of the coronary arteries due to the buildup of fatty proteins. Blood clots can obstruct normal blood flow, restricting the amount of nutrients and oxygen required for the heart to function properly. This may lead to heart attacks.

Another possibility is that the inflammation caused by periodontal disease increases plaque build up, which may contribute to swelling of the arteries.

Researchers have found that people with periodontal disease are almost twice as likely to suffer from coronary artery disease as those without periodontal disease. (American Academy of periodontology, 5)

The association between periodontitis and CAD may be because of common risk factors such as smoking,diabet,male gender and socioeconomic factors,but there is also good evidence of periodontitis being an independent risk factor for CAD.(2,15) Furthermore, periodontal pathogens have been identified in early as well as advanced atherosclerotic lesions.^16^ There is also some evidence that periodontitis is associated with increased plasma concentrations of pro-atherogenic Lipoproteins.^17^, ^18^ A study done by Buhlin K. And colleagues on the Range 143 women aged 43 to 79 years of age with CAD as a case group and 50 women 45 to 77 years old without CAD. OPG (Orthopanogram) were obtained for all patients and they were matched as viewpoints of other risk factors. The result of this study was, the women with CAD had lower oral and dental health conditions than women without CAD and there has been a significant relationship between periodontal disease and CAD. (19,20)

However multivariable analyses indicate that periodontal status is not significantly associated with CHD in either ever smokers or never smokers.

Clinical signs of periodontal disease were not associated with CAD, whereas systemic antibody response was associated with CAD in ever smokers and never smokers. These findings indicate that the quality and quantity of the host response to oral bacteria may be an exposure more relevant to systemic atherothrombotic coronary events than clinical measures.(21)

## References

[1] Wood D (2001). Established and emerging cardiovascular risk factors. Am Heart J.

[2] Villar F, Banegas JR, Rodri'guez F, Rey J (1998). Mortalidad de causa cardiovascular en Espan∼a y sus comunidades auto'nomas (1975–1992). Med Clin (Barc).

[3] Wilson PW, D'Agostino RB, Levy D, Belanger AM, Silbershatz H, Kannel WB (1998). Prediction of coronary heart disease using risk factor categories. Circulation.

[4] Beck J, Garcia R, Heiss G, Vokonas PS, Offenbacher S (1996). Periodontal disease and cardiovascular disease. J Periodontol.

[5] Loesche WJ, Schork A, Terpenning MS, Chen YM, Kerr C, Dominguez BL (1998). The relationship between dental disease and cerebral vascular accident in elderly United States veterans. Ann Periodontol.

[6] Hujoel PP (2002). Does chronic periodontitis cause coronary heart disease? A review of the literature. J Am Dent Assoc.

[7] Offenbacher S, Beck JD (2005). A perspective on the potential cardioprotective benefits of periodontal therapy. Am Heart J.

[8] Renvert S, Pettersson T, Ohlsson O, Persson GR (2006). Bacterial profile and burden of periodontal infection in subjects with a diagnosis of acute coronary syndrome. J Periodontol.

[9] Spahr A, Klein E, Khuseyinova N, Boeckh C, Muche R, Kunze M (2006). Periodontal infections and coronary heart disease: role of periodontal bacteria and importance of total pathogen burden in the Coronary Event and Periodontal Disease (CORODONT) study. Arch Intern Med.

[10] Ridker PM, Hennekens CH, Buring JE, Rifai N (2000). C-reactive protein and other markers of inflammation in the prediction of cardiovascular disease in women. N Engl J Med.

[11] DeStefano F, Anda RF, Kahn HS, Williamson DF, Russell CM (1993). Dental disease and risk of coronary heart disease and mortality. BMJ.

[12] Hujoel PP, Drangsholt M, Spiekerman C, DeRouen TA (2000). Periodontal disease and coronary heart disease risk. JAMA.

[13] Hujoel PP, Drangsholt M, Spiekerman C, DeRouen TA (2002). Pre-existing cardiovascular disease and periodontitis: a follow-up study. J Dent Res.

[14] Wu T, Trevisan M, Genco RJ, Dorn JP, Falkner KL, Sempos CT (2000). Periodontal disease and risk of cerebrovascular disease: the first national health and nutrition examination survey and its follow-up study. Arch Intern Med.

[15] Janket SJ, Baird AE, Chuang SK, Jones JA (2003). Meta-analysis of periodontal disease and risk of coronary heart disease and stroke. Oral Surg Oral Med Oral Pathol Oral Radiol Endod.

[16] Madianos PN, Bobetsis GA, Kinane DF (2002). Is periodontitis associated with an increased risk of coronary heart disease and preterm and/or low birth weight births?. J Clin Periodontol.

[17] Silness J, Loe H (1964). Periodontal disease inpregnancy. II. Correlation between oral hygiene and periodontal condtion. Acta Odontol Scand.

[18] Briggs JE, McKeown PP, Crawford VL, Woodside JV, Stout RW, Evans A (2006). Angiographically confirmed coronary heart disease and periodontal disease in middle-aged males. J Periodontol.

[19] Buhlin K, Gustafsson A, Ahnve S, Janszky I, Tabrizi F, Klinge B (2005). Oral health in women with coronary heart disease. J Periodontol.

[20] Lopez R, Oyarzun M, Naranjo C, Cumsille F, Ortiz M, Baelum V (2002). Coronary heart disease and periodontitis -- a case control study in Chilean adults. J Clin Periodontol.

[21] Fentoglu O, Bozkurt FY (2008). The Bi-Directional Relationship between Periodontal Disease and Hyperlipidemia. Eur J Dent.

[22] Beck JD, Eke P, Heiss G, Madianos P, Couper D, Lin D (2005). Periodontal disease and coronary heart disease: a reappraisal of the exposure. Circulation.

